# The Clinical Reasoning Mapping Exercise (CResME): a new tool for exploring clinical reasoning

**DOI:** 10.1007/s40037-018-0493-y

**Published:** 2019-01-21

**Authors:** Dario M. Torre, Caridad A. Hernandez, Analia Castiglioni, Steven J. Durning, Barbara J. Daley, Paul A. Hemmer, Jeffrey LaRochelle

**Affiliations:** 1 Uniformed Services University of Health Sciences, Bethesda, MD USA; 2 Medical School, University of Central Florida, Orlando, FL USA; 3 Department of Adult and Continuing eEducation, University of Wisconsin, Milwaukee, WI USA

**Keywords:** Clinical reasoning, Meaningful learning, Instruction, Assessment

## Abstract

**Introduction:**

National organizations have identified a need for the creation of novel approaches to teach clinical reasoning throughout medical education. The aim of this project was to develop, implement and evaluate a novel clinical reasoning mapping exercise (CResME).

**Methods:**

Participants included a convenience sample of first and second year medical students at two US medical schools: University of Central Florida (UCF) and Uniformed Services University of Health Sciences (USUHS). The authors describe the creation and implementation of the CResME. The CResME uses clinical information for multiple disease entities as nodes in different domains (history, physical exam, imaging, laboratory results, etc.), requiring learners to connect these nodes of information in an accurate and meaningful way to develop diagnostic and/or management plans in the process.

**Results:**

The majority of medical students at both institutions felt that the CResME promoted their understanding of the differential diagnosis and was a valuable tool to compare and contrast elements of a differential diagnosis. Students at both institutions recommended using the CResME for future sessions.

**Discussion:**

The CResME is a promising tool to foster students’ clinical reasoning early in medical school. Research is needed on the implementation of the CResME as an instructional and assessment strategy for clinical reasoning throughout medical school training.

**Electronic supplementary material:**

The online version of this article (10.1007/s40037-018-0493-y) contains supplementary material, which is available to authorized users.

## Introduction

Clinical reasoning is vitally important for practitioners across the health professions [1, 2]. In the report, ‘Improving Diagnosis in Health Care’, the National Academies of Sciences (NAS) asserts that improving the diagnostic process is a professional, moral and a public health obligation [3]. This call from the NAS requires that medical educators identify and develop effective instructional approaches linked to the sciences of learning that promote clinical reasoning across the educational continuum. However, it is not clear what strategies are most effective in promoting medical students’ clinical reasoning or how to optimally assess this essential ability [4]. Medical students most often practise clinical reasoning in the context of clinical rotations (i. e., clerkships). However, the limited number and type of patients available for practice, scattered supervision, and opportunities for feedback and reflection [5] limit the role of clinical rotations in learning clinical reasoning. A recent survey of US clerkship directors underscored the need to not only initiate clinical reasoning instruction earlier in the medical curriculum, but also provide consistent instruction across all years of training [6].

Schmidt and Mamede [7] posit that different clinical reasoning teaching strategies may be needed at different stages of medical students’ training. The addition of novel clinical reasoning strategies focused on eliciting learners’ thinking processes provides opportunities for meaningful feedback. These strategies can facilitate learners’ reflection and subsequent knowledge organization [8]. The purpose of the innovation was to develop, implement and evaluate a novel Clinical Reasoning Mapping Exercise (CResME, pronounced Kres-me) aimed at promoting clinical reasoning among pre-clerkship medical students.

### The CResME: theoretical frameworks and structure

The development of the Clinical Reasoning Mapping Exercise (CResME) instructional tool was informed by educational theory. Differentiating key features and comparing and contrasting related diagnoses are processes promoted by the CResME.

Assimilation theory of learning [9] makes a key distinction between rote learning and meaningful learning. Meaningful learning is related to previous knowledge and linked to an existing cognitive framework. The explicit exploration of meaningful relationships among ideas, the identification of similarities and differences across ideas, and the reconciliation of inconsistencies allows the learner to delineate similarities and differences between concepts.

Illness scripts represent organized knowledge structures [8] that include prototypical features (signs and symptoms, etc.) of a disease interconnected by causal relationships, that experienced physicians are believed to use in practice [10, 11]. Linking separate prototypical features are crucial steps in the development of illness scripts. Instructional tools that engage the learner in the process of comparing and contrasting multiple diagnoses may enhance meaningful learning and the development of more sophisticated illness scripts [12].

The use of CResMEs in groups is consistent with social cognitive theories (i. e. situated learning) which argue that learning is a group process that is the result of interactions with others as well as artifacts in the environment and that learning entails legitimate participation as opposed to acquisition of static information [13]. Through providing a scaffold for group discussion, CResMEs can also promote peer learning.

## Methods

The CResME presents clinical information for multiple disease entities as nodes (boxes) in different domains (history, laboratories, etc.), requiring learners to connect these nodes of information in an accurate and meaningful way (see Fig. 1a of the online electronic supplementary material). A brief description with a chief complaint is listed at the top of the sheet. The nodes (boxes) are vertically structured and contain prototypical clusters of information related to several potential diagnoses for a particular patient presentation. The nodes are randomly placed and learners create links by making connections among the nodes and writing a final diagnosis in an empty node, as depicted in Fig. 1b of the online material.

The CResME can be modified for content, structure, level of difficulty, and learners’ tasks. For example, the difficulty can be increased by including more nodes than are needed or by leaving one or more nodes empty. Images (e. g. X‑rays), heart sounds, and/or pathological findings can be integrated either in paper or an electronic format.

We implemented the CResME at two medical schools: the University of Central Florida College of Medicine (UCF-COM) and Uniformed Services University of Health Sciences (USUHS). At UCF-COM the CResME activity was introduced in the Practice of Medicine second-year course. The first CResME activity was scheduled for a faculty-led two-hour session (M2; *n* = 92/113). At USUHS, the CResME was introduced at the beginning of year one of medical school (M1; *n* = 93/160) in the Introduction to Clinical Reasoning course. The USUHS CResME activity was a 30-minute preceptor-led post Standardized Patient encounter session. At USUHS, faculty use of the CResME was voluntary, thus a small number of students did not participate in the activity.

For this CResME at UCF-COM, students were presented with a brief description of a patient with chest pain in an electronic format, and instructed to consider four different diseases. Through comparing and contrasting, students would link the history of the present illness with the expected physical exam, appropriate diagnostic workup findings, and ultimately identify final diagnoses (see Fig. 1 of the online material). Learners worked in groups of 5–6 per table and there were four groups of students per faculty facilitator. Learners were instructed to complete the chest pain CResME independently. Following this, learners discussed and compared their individual CResMEs with the group, and then agreed on a group version. Finally, faculty led the debriefing, which included learners presenting how they arrived at their final diagnoses.

At USUHS the CResME provided medical students with an opportunity to identify, compare and contrast clinical components of a differential diagnosis after they completed three standardized patient encounters of low back pain (lumbago, herniated disk, and vertebral osteomyelitis). The CResME content was based on these three cases with the addition of a fourth diagnosis (spinal cord compression syndrome). Learners completed the CResME in groups of six during a 30-minute faculty led debriefing session. Each group began solving the exercise, sharing their thinking and rationale with the group. Faculty facilitated the session by asking questions and eliciting learners’ thinking about key features emerging from the exercise.

The content of the exercises was developed by a group of experienced clinicians at both institutions and any disagreement was resolved by discussion until consensus was achieved. At both institutions, teachers were provided with a Faculty Guide, which included a completed CResME map, key teaching points, and instructions on how to conduct the session. Students also received verbal and written instructions on how to complete the CResME before each session.

At both institutions, a 5-item evaluation form was completed by students after each session (*n* = 93 at USUHS; *n* = 92 at UCF-COM). Students were asked whether the CResME promoted their understanding of the differential diagnosis and whether it was a valuable tool to compare and contrast elements of the differential diagnosis. Students were also queried if they would recommend using the tool for future sessions and what suggestions they had for improvement. Descriptive statistics were performed and a qualitative analysis was completed from the open-ended questions. The authors reviewed the comments independently, coded into categories and reduced into themes. Discussions were held to until complete consensus was achieved. The project was approved by the Institutional Review Board at both institutions.

## Results

Medical students at both institutions felt that the CResME promoted their understanding of the differential diagnosis, was a valuable tool to compare and contrast elements of a differential diagnosis and recommended using the CResME for future sessions (Tab. 1).

**Table 1:** A comparison of CResME’s impact on USUHS and UCF medical students

	USUHS *n* (93/160)	UCF *n* (92/113)
Medical students (year)	MS1	MS2
Setting	Post simulated encounter debriefing session	Stand-alone session within teaching module
Topics of the CResME	Low back pain, abdominal pain	Low back pain, abdominal pain
Evaluation (mean, SD)	Mean (SD)	Mean (SD)
Promote understanding of differential diagnosis	4.42 (0.66)	4.33 (0.65)
Valuable to compare and contrast elements of differential diagnosis	4.40 (0.66)	4.30 (0.65)
	*n* (%)	*n* (%)
Recommended using the CResME for future sessions	88 (95%)	82 (90%)
Faculty led session	yes	yes
Format	Paper	Electronic

Likert scale (1 = strongly disagree; 5 = strongly agree)

The following two themes were identified: use of the CResME as a tool to promote their thinking about a differential diagnosis through comparing and contrasting, and the role of the CResME in facilitating group learning and discussion. Students found the process of comparing and contrasting clinical features of different diagnoses particularly valuable. One student stated ‘*the exercise helped me identify differences among similar diagnoses’*, another reported ‘*I like being able to compare sets of history and PE [physical exam] findings side by side in order to better see the similarities and differences between diagnoses to see how they relate or differ.*’ Students indicated that the exercise allowed for interaction and discussion with peers and faculty. One student stated ‘*the exercise allowed for group discussion of differential diagnosis and explanation of how we can tell them apart’*. Finally, students provided comments on improving the tool. One student indicated ‘*more HPI [history of the present illness] information would be helpful’*. Faculty commented that the CResME provided a helpful scaffold to teach clinical reasoning and that learners enjoyed the exercise as it made implicit features of clinical reasoning explicit to both the learner and the teacher. Some faculty stated that they plan to use the CResME in future clinical reasoning teaching sessions with medical students and residents.

## Discussion

Our findings suggest that the CResME is valuable as a clinical reasoning learning tool and recommend its use for future sessions. The CResME holds promise as a powerful tool to teach clinical reasoning to a range of learners. We have shown the flexibility of this tool across learners at different schools and levels of training using both paper and electronic formats, across a variety of content areas. Further, this instructional approach could be used to foster the integration of basic and clinical sciences.

There are two clinical reasoning tools that have been previously described in the literature to foster development of illness scripts: the Clinical Integrative Puzzle (CIP) [14, 15] and the MATCH test [16]. However the CResME differs in four major ways. First, with the CResME students are tasked to provide a final diagnosis for each included disease based on a correct integration and processing of the prototypical features of each disease presented in the nodes versus being given the diagnoses with the CIP and MATCH test. Second, the visual representation and alignment of the nodes next to each other is unique with the CResME. Third, a node can be used once, more than once or not at all (versus the one-to-one matching with the CIP and MATCH). Finally, the CResME allows multiple opportunities to increase the degree of difficulty of the exercise such as adding distractor nodes, leaving empty nodes to fill in, integrating multimedia which are not described with the CIP or MATCH test.

Future research, using qualitative methods and think aloud protocols [4], should focus on using the CResME as a scaffold to further our understanding of learners’ clinical reasoning. It would also be of interest to determine how a student solves a CResME to elucidate the reasons behind a chosen thinking strategy. Additionally, gathering validity evidence on this instrument would be of interest.

The study has several limitations. First, the development of multiple CResMEs on different topics may be faculty intensive. Faculty reported taking approximately 1–3 hours to develop a CResME. However, once a CResME has been developed, changes can easily be made, allowing the creation of different versions of the CResME without a significant time investment. Second, evidence that the CResME enhances learning clinical reasoning by students needs to be gathered. Third, we did not collect data as to why some faculty at USUHS did not use the tool. Finally, faculty development will be needed to maximize students’ learning of clinical reasoning at different stages of training, and how to develop CResMEs that allow full integration of basic and clinical sciences. Indeed, orienting faculty and learners to the CResME is essential for successful teaching sessions.

Educators can also create a series of CResMEs with variable levels of difficulty that can be used with different learners (early, mid, or late medical students, interns, residents, fellows, or even practicing clinicians) throughout the entire medical education continuum, in different settings (simulation, clinical setting) and formats (electronic vs. paper). Finally, this instructional tool can become, along with other teaching strategies, an integral part of a clinical reasoning teaching and assessment toolbox for medical educators.

## Caption Electronic Supplementary Material

Figure series of CResMEs on Chest Pain

## References

[1] Eva KW (2005). What every teacher needs to know about clinical reasoning. Med Educ.

[2] Trowbridge RL, Rencic J, Durning SJ (2015). Teaching clinical reasoning.

[3] Committee on Diagnostic Error in Health Care, Board on Health Care Services, Institute of Medicine, The National Academies of Sciences, Engineering, and Medicine (2015). Improving diagnosis in health care.

[4] Kassirer JP (2010). Teaching clinical reasoning: case-based and coached. Acad Med.

[5] Wimmers PF, Schmidt HG, Splinter TA (2006). Influence of clerkship experiences on clinical competence. Med Educ.

[6] Rencic J, Trowbridge RL, Fagan M (2017). Clinical reasoning education at US medical schools: results from a national survey of internal medicine clerkship directors. J Gen Intern Med.

[7] Schmidt HG, Mamede S (2015). How to improve the teaching of clinical reasoning: a narrative review and a proposal. Med Educ.

[8] Bordage G (1994). Elaborated knowledge: a key to successful diagnostic thinking. Acad Med.

[9] Ausubel DP (2000). The acquisition and retention of knowledge: a cognitive view.

[10] Schank RC, Abelson RP (2013). Scripts, plans, goals, and understanding: an inquiry into human knowledge structures.

[11] Charlin B, Boshuizen HP, Custers EJ (2007). Scripts and clinical reasoning. Med Educ.

[12] Schmidt HG, Rikers RM (2007). How expertise develops in medicine: knowledge encapsulation and illness script formation. Med Educ.

[13] Cleland J, Durning SJ (2015). Researching medical education.

[14] Ber R (2003). The CIP (comprehensive integrative puzzle) assessment method. Med Teach.

[15] Capaldi VF, Durning SJ, Pangaro LN, Ber R (2015). The clinical integrative puzzle for teaching and assessing clinical reasoning: preliminary feasibility, reliability, and validity evidence. Mil Med.

[16] Groothoff JW, Frenkel J, Tytgat GAM, Vreede WB, Bosman DK, ten Cate OTJ (2008). Growth of analytical thinking skills over time as measured with the MATCH test. Med Educ.

