# Supplementary material for: The Clinical Reasoning Mapping Exercise (CResME): a new tool for exploring clinical reasoning
Source: Perspect Med Educ. 2019 Jan 21;8(1):47–51. doi: 10.1007/s40037-018-0493-y (PMC6382623; doi:10.1007/s40037-018-0493-y)
Supplement: Supplementary file 1 — Figure series of CResMEs on Chest Pain [file 40037_2018_493_MOESM1_ESM.docx]

Fig 1a: CResME for chest pain example

**55 yo with chest pain**

|  |  |  |  |  |
| --- | --- | --- | --- | --- |
| **HPI** | **A**  **B**  Acute retrosternal chest pain; squeezing sensation; present for 6 hours. Doesn't seem to radiate | **C**  Acute chest pain; retrosternal and radiates to upper back/left scapular region and downwards. Onset 90 minutes ago.  Acute retrosternal chest pain/- pressure; severe, persistent; radiates to neck/ left shoulder.  Onset 45 minutes ago. | **D** | Acute chest pain; sharp, retrosternal; radiates to neck and varies somewhat with position. Recent febrile illness.  Onset 60 min ago |
| **PHYSICAL EXAM**  **E**  T:38.2 BP:112/60 HR:110 RR:18  Gen: uncomfortable but NAD  CV: Reg rate; NI S1, S2, systolic -diastolic sound over the precordium  Lungs: CTA, b/I  Ext: No edema;  Radial pulses equal | **F**  T:37.2 BP:180/100 HR:120  RR:18  Gen: Tall. slim man; distressed CV:RRR; NI S1,S2;Gr2/6 decrescendo diastolic murmur, LLSB  Lungs: CTA, b/I  Ext: No edema;  Radial pulses R>L | **G** | **H**  37.0 BP:100/72 HR:90  RR:16  Gen: distressed, diaphoretic CV:RRR; NL S1,S2;No +S4  Lungs: CTA, b/I  Ext: No edema;  Radial pulses equal  T:37.0 BP:138/72 HR:76  RR:16  Gen: Uncomfortable but NAD CV:RRR; NL S1,S2;No S3/S4  Lungs: CTA, b/I  Ext: No edema;  Radial pulses equal |  |
| **DATA**  **I**  WBC: 15,000  CXR: Normal  ECG: Sinus tachycardia; diffuse concave ST segment elevation and PR segment depression  Troponins: normal | **J**  WBC: 9,000  CXR: Normal  ECG: normal  Troponins: normal | **K**  WBC: 8,000  CXR: Normal  ECG: NSR, ST depression and T wave inversions V4-V6  Troponins: elevated | **L**  WBC: 15,000  CXR: widened mediastinum  ECG: sinus tachycardia, LVH  Troponins: normal |  |
| **DIAGNOSIS**  **M** | *Write in Diagnosis*  **N**  *Write in Diagnosis* | *Write in Diagnosis*  **O** |  | *Write in Diagnosis*  **P** |

The CResME presents clinical information for multiple disease entities with a common presentation as nodes (boxes) in different domains. Learners connect these nodes of information in a meaningful way to develop an illness script for each diagnosis the learner writes in. The nodes are vertically structured in a random fashion and contain prototypical clusters of information related to a series of specific diagnoses. Learners arrange the nodes in the correct sequence to develop an accurate illness script for the diagnosis they write into the blank box at the bottom.

Figure 1b: How Learner May Navigate CResME

**55 yo with chest pain**

|  |  |  |  |  |
| --- | --- | --- | --- | --- |
| **HPI** | **A**  Acute retrosternal chest pain; squeezing sensation; present for 6 hours. Doesn't seem to radiate | **C**  Acute chest pain; retrosternal and radiates to upper back/left scapular region and downwards. Onset 90 minutes ago.  Acute retrosternal chest pain/- pressure; severe, persistent; radiates to neck/ left shoulder.  Onset 45 minutes ago. | **D** | Acute chest pain; sharp, retrosternal; radiates to neck and varies somewhat with position. Recent febrile illness.  Onset 60 min ago |
| **PHYSICAL**  **E**  T:38.2 BP:112/60 HR:110 RR:18  Gen: uncomfortable but NAD  CV: Reg rate; NI S1, S2, systolic-diastolic sound over the precordium  Lungs: CTA, b/I  Ext: No edema;  Radial pulses equal | **F**  T:37.2 BP:180/100 HR:120  RR:18  Gen: Tall. slim man; distressed CV:RRR; NI S1,S2;Gr2/6 decrescendo diastolic murmur, LLSB  Lungs: CTA, b/I  Ext: No edema;  Radial pulses R>L | **B**  **G** | **H**  37.0 BP:110/72 HR:90  RR:16  Gen: distressed, diaphoretic CV:RRR; NL S1,S2;No +S4  Lungs: CTA, b/I  Ext: No edema;  Radial pulses equal  T:37.0 BP:138/72 HR:76  RR:16  Gen: Uncomfortable but NAD CV:RRR; NL S1,S2;No S3/S4  Lungs: CTA, b/I  Ext: No edema;  Radial pulses equal |  |
| **DATA**  **I**  WBC: 15,000  CXR: Normal  ECG: Sinus tachycardia; diffuse concave ST segment elevation and PR segment depression  Troponins: normal | **J**  WBC: 9,000  CXR: Normal  ECG: normal  Troponins: normal | **K**  WBC: 8,000  CXR: Normal  ECG: NSR, ST depression and T wave inversions V4-V6  Troponins: elevated | **L**  WBC: 15,000  CXR: widened mediastinum  ECG: sinus tachycardia, LVH  Troponins: normal |  |
| **DIAGNOSIS**  **M** | *Write in Diagnosis*  **N**  *Write in Diagnosis* | **NSTEMI**  **O** |  | *Write in Diagnosis*  **P** |

A learner may first review, process, compare and contrast the information contained in each of the history of present illness (HPI nodes). For example, learners may focus first on the historical features of acute coronary syndrome (ACS/NSTEMI), node B. Then they move vertically looking for the physical exam (PE) findings that would be expected in a patient with ACS. They continue to compare and contrast findings within and across each node, eliminating other PE nodes based on discriminating features. Therefore, they connect history findings of NSTEMI, node B, with the most likely PE findings associated with NSTEMI( Non-ST elevation myocardial infarction), node H. Next, based on the previous combination of HPI and PE, the learner moves to examine the diagnostic data presented in the data nodes. The learner continues to compare, contrast, and differentiate within and across data nodes, using a single (e.g, WBC) or a combination of diagnostic data (WBC, CXR, EKG etc.) findings to discriminate across diagnoses. Next, the learner looks to accurately connect HPI and PE pair consistent with a preliminary diagnosis of NSTEMI to the appropriate data findings. In the data section, the learner is presented with the challenge of interpreting and integrating three diagnostic studies, two electrocardiograms (ECG) and one chest radiograph (CXR). The learner recognizes troponin elevation and the ECG findings of significant ST depression in leads V3-V5, as diagnostic findings most consistent with ACS/NSTEMI and makes another connection, node K. At the end, the learner writes the most likely diagnosis, NSTEMI in the empty box of, as a result of the integration all the information from HPI, PE and diagnostic data. The learner would then continue with the exercise, processing, comparing and contrasting cluster of information within and across nodes of different domains, making the appropriate connections to generate and write a diagnosis for each presentation and cluster of nodes. The exercise is completed when all comparisons are checked and completed, all the nodes accurately linked, and four diagnoses are generated and written in the empty nodes at the bottom of the exercise. An answer sheet can be provided to learners at the end of the exercise.

Here is the **answer key** for this exercise: Non-ST Elevation myocardial infarction: B, H, K O; Gastroesophageal reflux disease(GERD): A, G, J, M; acute pericarditis: D,E,I,M; aortic dissection: C,F,L,P.
